# Composition, Cytotoxic and Antimicrobial Activities of *Satureja intermedia* C.A.Mey Essential Oil

**DOI:** 10.3390/ijms160817812

**Published:** 2015-08-03

**Authors:** Javad Sharifi-Rad, Mehdi Sharifi-Rad, Seyedeh Mahsan Hoseini-Alfatemi, Marcello Iriti, Majid Sharifi-Rad, Marzieh Sharifi-Rad

**Affiliations:** 1 Zabol Medicinal Plants Research Center, Zabol University of Medical Sciences, Zabol 61615-585, Iran; E-Mail: javad.sharifirad@gmail.com; 2 Department of Pharmacognosy, Faculty of Pharmacy, Zabol University of Medical Sciences, Zabol 61615-585, Iran; 3 Department of Medical Parasitology, Zabol University of Medical Sciences, Zabol 61663-335, Iran; E-Mail: mehdi_sharifirad@yahoo.com; 4 Pediatric Infections Research Center, Mofid Children Hospital, Shahid Beheshti University of Medical Sciences, Tehran 15468-15514, Iran; 5 Department of Agricultural and Environmental Sciences, Milan State University, via G. Celoria 2, Milan 20133, Italy; 6 Department of Range and Watershed Management, Faculty of Natural Resources, University of Zabol, Zabol 98615-538, Iran; E-Mail: majid.sharifirad@gmail.com; 7 Department of Chemistry, Faculty of Science, University of Zabol, Zabol 98615-538, Iran; E-Mail: marzieh.sharifirad@gmail.com

**Keywords:** GC/MS, oral pathogens, human cancer cells, hepatocellular carcinoma, breast adenocarcinoma

## Abstract

In this study, the essential oil (EO) constituents from the aerial parts of *Satureja intermedia* C.A.Mey were detected by GC and GC/MS. The antimicrobial activity of EO on oral pathogens and its cytotoxicity to human cancer cells were determined by the microbroth dilution method and the crystal violet staining method, respectively. Thirty-nine compounds were identified and the main EO constituents were γ-terpinene (37.1%), thymol (30.2%), *p*-cymene (16.2%), limonene (3.9%), α-terpinene (3.3%), myrcene (2.5%), germacrene B (1.4%), elemicine (1.1%) and carvacrol (0.5%). The *S. intermedia* EO showed a concentration-dependent decrease in viability of Hep-G2 (hepatocellular carcinoma) and MCF-7 (breast adenocarcinoma) human cancer cell lines (*p* < 0.05). Antimicrobial screening of *S. intermedia* EO demonstrated slight antibacterial and antifungal activities against *Streptococcus mutants*, *S. salivarius*, *Enterococcus faecalis*, *Staphylococcus aureus*, *Candida albicans* and *C. glabrata.* Further preclinical studies are needed to assess the efficacy and safety of *S. intermedia* EO as a new promising anticancer agent.

## 1. Introduction

Nowadays, many classes of synthetic pharmaceuticals are prescribed in conventional medicine for disease treatment and management. However, in case of infectious diseases, these therapies are showing a reduced efficacy because of the emergence of drug-resistant microbial strains [1,2,3]. Similarly, the failure of chemotherapy used in cancer treatment can be due to the phenomenon of multidrug resistance in cancer cells [4,5]. Therefore, the use of phytotherapeutics, which, in general, show fewer adverse effects than conventional drugs, represents a promising anticancer strategy as well as an attractive approach to control infections and contaminations in medicine, veterinary, phytoiatry and food sciences [6,7,8,9].

Dental caries (tooth decay) is a multi-factorial disease due to the demineralization of the hard dental tissues (enamel, dentin and cementum) caused by the bacterial plaque (bacterial biofilm) that covers the tooth surface [10,11]. It has been estimated that tooth decay affects about 3.9 billion people throughout the world, and untreated caries in permanent teeth is the most common condition in countries with low socio-economic status [12].

Twenty-five species of oral streptococci live the human oral cavity, representing about 20% of the total oral bacteria. In addition, yeast species are found in oral cavity as a normal microbial flora [13]. Oral streptococci are involved in tooth decay and a number of oral infections. Yeast species may colonize and adhere to soft and hard tissue surfaces, and form a biofilm when immunosuppressive agents or broad-spectrum antibiotics are prescribed to dental patients.

As previously introduced, the emergence of antibiotic resistance and multidrug resistance in microbial and cancer cells, respectively, may seriously threaten the success of conventional therapies, thus opening a new and alternative scenario which arises from the traditional herbal medicines [14,15,16,17,18,19,20].

Essential oils (EOs) are complex mixtures of lipophilic, volatile and aromatic plant secondary metabolites [21]. *Satureja intermedia* C.A.Mey, belonging to *Lamiaceae* family, is a medicinal plant native to Mediterranean region and Iran. The aerial parts of *Satureja* species have a typical taste and can be added to meat, pies, stuffing and sausages as a spice. These species owing to their stimulating, tonic and carminative effect, are also used as a herbal tea and seasoning [22]. In previous studies, the EO composition and antimicrobial activity of some *Satureja* species have been reported [23,24,25,26,27,28]. In addition, the EOs extracted from *Satureja* species, which differ in both morphological and phytochemical traits [29], showed several biological properties such as antioxidant [30], anti-inflammatory and analgesic [31], antifungal and antibacterial [27,32], antispasmodic and anti-diarrheal [32], anti-hyperlipidemic, reproduction-stimulatory and antidiabetic activities [33]. *Satureja* spp. EOs also improved fertility, stomach disorders, thrombosis, cardiovascular diseases [34,35,36] and relieved intestinal and muscle pain.

In this study, the EO constituents from the aerial parts of *Satureja intermedia* C.A.Mey were detected, and the antimicrobial and cytotoxic activities of EO were determined on oral pathogens and human cancer cells, respectively.

## 2. Results and Discussion

### 2.1. Chemical Composition of S. intermedia Essential Oil

The chemical composition of *S. intermedia* EO is shown in Table 1.

**Table 1:** Monoterpene and sesquiterpene constituents of *Satureja intermedia* C.A.Mey essential oil (EO).

Compound	RI *	Relative (%)
α-Thujene	933	0.3
α-Pinene	937	0.2
Camphene	955	0.3
β-Pinene	983	0.5
Myrcene	990	2.5
3-Octanol	1003	t
α-Phellandrene	1008	0.4
δ-3-Carene	1010	t
α-Terpinene	1020	3.3
*p*-Cymene	1024	16.2
Limonene	1029	3.9
1,8-Cineole	1036	t
β-Ocimene	1049	0.1
γ-Terpinene	1056	37.1
*cis*-Sabinene hydrate	1065	t
Terpinolene	1087	t
*trans*-Sabinene hydrate	1099	t
Linalool	1103	0.2
β-Fenchol	1120	0.1
Ipsdienol	1162	t
Borneol	1165	t
Terpinen-4-ol	1176	0.3
*p*-Cymen-8-ol	1181	t
α-Terpineol	1189	0.2
Thymoquinone	1245	0.1
Thymol	1290	30.2
Carvacrol	1295	0.5
Thymyl acetate	1342	0.1
β-Caryophyllene	1412	0.2
Germacrene D	1475	0.2
β-Bisabolene	1501	t
Viridiflorene	1510	0.1
δ-Cadinene	1523	0.1
Elemicine	1552	1.1
Germacrene B	1556	1.4
*cis*-Sesquisabinene hydrate	1564	t
Spathulenol	1577	t
Globulol	1582	t
α-Cadinol	1650	t
Monoterpene hydrocarbons		64.8
Oxygenated monoterpenes		31.6
Sesquiterpene hydrocarbons		3.1
Oxygenated sesquiterpenes		0.08
Others		0.1
Total identified		99.8%

* RI: retention index; t = traces (>0.05%).

In total, 39 compounds were identified, representing 99.8% of *S. intermedia* EO. The main constituents were γ-terpinene (37.1%), thymol (30.2%), *p*-cymene (16.2%), limonene (3.9%), α-terpinene (3.3%), myrcene (2.5%), germacrene B (1.4%), elemicine (1.1%) and carvacrol (0.5%).

On the basis of their major components, *Satureja* oils can be assigned to one of three main chemotypes: aromatic *p*-menthane monoterpenes, mainly carvacrol, thymol and *p*-cymene (chemotype I); aliphatic *p*-menthane monoterpenes, mainly menthone, isomenthone, pulegone and piperitone (chemotype II); or various mono- and sesquiterpenes (chemotype III) [37]. Sefidkon and Jamzad [26] analyzed the EO of the aerial parts of *S. intermedia* collected from the Ardebil provinces in Iran at the full flowering stage. The main components of the EO were 38 compounds including thymol (32.3%), γ-terpinene (29.3%) and *p*-cymene (14.7%) as the most abundant constituents. Sadeghi *et al.* [28] investigated the *S. intermedia* EO from Talesh, Iran, at full flowering stage; they detected thymol (34.5%), γ-terpinene (18.2%) and *p*-cymene (10.5%) as the major compounds. Our results are in agreement with these studies: no significant qualitative difference was observed in the *S. intermedia* EO composition, whereas quantitative differences may be due to ecological, environmental and genetic factors [6].

### 2.2. Cytotoxicity and Antimicrobial Activities

The results on cytotoxicity, antibacterial and antifungal assays are summarized in Table 2, Table 3 and Table 4, respectively. *S. intermedia* EO determined a significant dose-dependent decrease in viability of both cancer cell lines (*p* < 0.05) (Table 2). In case of Hep-G2 cells, IC_50_ of *S. intermedia* EO (IC_50_ ≥ 50 µg/mL) was higher than that of the vinblastine (IC_50_ = 6.65 μg/mL). Similarly, IC_50_ of the *S. intermedia* EO (IC_50_ ≥ 50 µg/mL) on MCF-7 was higher than that of the reference drug used (IC_50_ = 6.25 μg/mL). Therefore, the results showed a low cytotoxic activity of the *S. intermedia* EO on these cancer cells.

**Table 2:** Cytotoxic activity of *Satureja intermedia* C.A.Mey EO on human cancer cell lines.

Concentrations (µg/mL)	*S. intermedia* EO		Vinblastine	
	% Viability			
	Hep-G2 ^a^	MCF-7 ^b^	Hep-G2	MCF-7
0	100	100	100	100
1.56	89.33 ± 5.25	96.11 ± 2.01	74.35 ± 2.33	59.43 ± 0.22
3.125	81.11 ± 7.09	92.43 ± 2.11	58.33 ± 1.14	57.22 ± 1.11
6.25	78.83 ± 9.11	86.77 ± 4.29	51.14 ± 0.1	50.01 ± 3.00
12.5	70.13 ± 7.01	82.56 ± 4.61	38.54 ± 1.78	40.22 ± 4.17
25	66.55 ± 3.19	79.12 ± 7.61	11.19 ± 4.12	14.22 ± 1.01
50	64.11 ± 2.00	76.22 ± 2.11	9.22 ± 1.11	9.79 ± 2.17
IC_50_ *	>50 µg/mL	>50 µg/mL	6.65 µg/mL	6.25 µg/mL

* IC_50_: the sample concentration required to inhibit cancer cell proliferation by 50%; ^a^ Hep-G2: human hepatocellular carcinoma cells; ^b^ MCF-7: human breast adenocarcinoma cells; data are expressed as means ± SD.

**Table 3:** Antibacterial activity of *Satureja intermedia* C.A.Mey EO.

Microorganisms	*S. intermedia* EO		Vancomycin ^4^	
	MIC ^2^ (mg/mL)	MBC ^3^ (mg/mL)	MIC (mg/mL)	MBC (mg/mL)
*Streptococcus mutants *(ATCC 25175)	4.2 ± 0.0 ^1^	20 ± 0.0	0.0025	0.014 ± 0.0
*S. mutants *(ATCC 31383)	4.6 ± 0.3	20 ± 0.0	0.0025	0.014 ± 0.0
*S. mutants *(ATCC 35668)	5.1 ± 0.0	20 ± 0.0	0.0025	0.014 ± 0.0
*Streptococcus salivarius *(ATCC 13419)	12.5 ± 0.2	38.4 ± 0.2	0.044	0.065 ± 0.0
*S. salivarius *(ATCC 9222)	11.4 ± 0.0	36.5 ± 0.0	0.044	0.065 ± 0.0
*Enterococcus faecalis *(ATCC 29212)	8.9 ± 0.0	25.5 ± 0.3	0.036	0.045 ± 0.0
*E. faecalis *(ATCC11700)	9.2 ± 0.6	25.2 ± 0.1	0.036	0.045 ± 0.0
*Staphylococcus aureus *(ATCC 25923)	6.5 ± 0.0	19.4 ± 0.1	0.012	0.011 ± 0.0
*S. aureus *(ATCC 700698)	7.7 ± 0.4	20.4 ± 0.1	0.012	0.011 ± 0.0

^1^ Data are expressed as mean ± SD of ^2^ MIC (minimum inhibitory concentration) and ^3^ MBC (minimum bactericidal concentration) of *S. intermedia* EO and ^4^ reference drug (vancomycin).

**Table 4:** Antifungal activity of *Satureja intermedia* C.A.Mey essential oil (EO).

Microorganisms	*S. intermedia* EO		Ketoconazole ^4^	
	MIC ^2^ (mg/mL)	MFC ^3^ (mg/mL)	MIC (mg/mL)	MFC (mg/mL)
*Candida albicans *(ATCC 13803)	3.4 ± 0.2 ^1^	7.5 ± 0.4	0.0039 ± 0.0	0.0068 ± 0.0
*C. albicans *(ATCC 10261)	4.2 ± 0.0	8.33 ± 0.3	0.0039 ± 0.0	0.0068 ± 0.0
*Candida glabrata *(ATCC 2001)	3.5 ± 0.1	6.8 ± 0.5	0.0039 ± 0.0	0.0068 ± 0.0
*C. glabrata *(ATCC 90030)	3.8 ± 0.3	5.2 ± 0.2	0.0039 ± 0.0	0.0068 ± 0.0

^1^ Data are expressed as mean ± SD of ^2^ MIC (minimum inhibitory concentration) and ^3^ MFC (minimum fungicidal concentration) of *S. intermedia* EO and ^4^ reference drug (ketoconazole).

Sadeghi *et al.* [28] investigated the cytotoxicity of *S. intermedia* EO on the human bladder carcinoma and esophagus squamous cell carcinoma cell lines. They reported an IC_50_ value of 156 µg/mL and suggested that *S. intermedia* EO may be applied as a potential anticancer agent. However, EOs cannot substitute the standard chemotherapy, even if they may play a role as adjuvant agents, potentiating the efficacy of conventional chemotherapeutics and decreasing their adverse effects.

The *S. intermedia* EO tested exhibited antibacterial and antifungal activities (Table 3 and Table 4). The minimum inhibitory concentration (MIC) for *S. mutants* strains ranged from 4.2 to 5.1 mg/mL. The minimum bactericidal concentration (MBC) values were 20 mg/mL for all *S. mutants* strains*.* The MICs and MBCs for vancomycin on all strains of *S. mutants* were 0.0025 and 0.014 mg/mL, respectively. The MICs for *S. salivarius* (ATCC 13419) and *S. salivarius* (ATCC 9222) were 12.5 and 11.4 mg/mL, respectively. MBC values were 38.4 and 36.5 mg/mL for *S. salivarius* (ATCC 13419) and *S. salivarius* (ATCC 9222), respectively*.* The MICs and MBCs for vancomycin on all strains of *S. salivarius* were 0.044 and 0.065 mg/mL, respectively. The MICs for *E. faecalis* (ATCC 29212) and *E. faecalis* (ATCC 11700) were 8.9 and 9.2 mg/mL, respectively. In addition, the MBC values were 25.5 and 25.2 mg/mL for these strains, respectively*.* The MICs and MBCs for vancomycin on all strains of *E. faecalis* were 0.036 and 0.045 mg/mL, respectively. The MICs for *S. aureus* (ATCC 25923) and *S. aureus* (ATCC 700698) were 6.5 and 7.7 mg/mL, respectively. The MBC values were 19.4 and 20.4 mg/mL for these strains, respectively*.* The MICs and MBCs for vancomycin on these strains of *S. aureus* were 0.012 and 0.011 mg/mL, respectively.

The MICs for all the tested *Candida* ranged from 3.4 to 4.2 mg/mL. Minimum fungicidal concentration (MFC) values were 7.5, 8.33, 6.8 and 5.2 mg/mL for *C. albicans* (ATCC 13803), *C. albicans* (ATCC 10261), *C. glabrata* (ATCC 2001) and *C. glabrata* (ATCC 90030), respectively. The MICs and MFCs for ketoconazole, on all *Candida* strains, were 0.0039 and 0.0068 mg/mL, respectively.

The antimicrobial activity of *Satureja* species has been recently emphasized [38]. Giweli *et al.* [39] investigated the antimicrobial activity of *S. thymbra* EO growing wild in Libya against eight bacterial and eight fungal species. This EO was highly effective against the microorganisms tested, particularly against the fungi. The *S. thymbra* EO showed bacteriostatic activity at 0.001–0.1 mg/mL and was bactericidal at 0.002–0.2 mg/mL; fungistatic and fungicidal effects were observed at 0.001–0.025 mg/mL and 0.001–0.1 mg/mL, respectively. The main *S. thymbra* EO components, thymol, carvacrol and γ-terpinene, also found in our *S. intermedia* EO, showed high antimicrobial activity.

Antimicrobial activity of the methanol extract of *S. montana* aerial parts was recently demonstrated on *S aureus*, *C. albicans* and *C. glabrata*. [40]. Similarly, *S. khuzestaica* and *S. bachtiarica* EOs inhibited the growth of oral pathogens, particularly of *E. faecalis* [41], as well as *S. bachtiarica* EO was also active against *Helicobacter pylori* [42]. Intriguingly, carvacrol, the main component of *S. bachtiarica* EO, exhibited a significant anti-*Helicobacter pylori* activity, whereas, in the presence of thymol, the antibacterial effect of carvacrol was reduced [42].

Oyedemi *et al.* [43] studied the mechanism of antimicrobial activity of the essential oil constituent’s α-terpineol, γ-terpinene and eugenol, evaluating their effect on the cell membrane of four bacterial strains. The results on lipid leakage showed that these compounds were effective against both Gram-positive and Gram-negative bacteria, damaging both the cell wall and the membrane.

Monoterpenes are lipophilic, volatile phytochemicals arising from isoprene (2-methyl-1,3-butadiene) and originated by the condensation of two isoprene units [44]. Among monoterpenes, thymol is well-known as an effective antifungal and antimicrobial agent. Due to its hydrophobicity, thymol may alter the structural and functional integrity of microbial cell membrane [44]. In addition, this compound may impair the adhesiveness and biofilm formation of fungi and bacteria [45]. The biocidal activity of *p*-cymene, a main component of *S. intermedia* EO, on both spoilage yeasts and bacteria has also been previously reported in previous study [46]. Limonene is another compound significantly present in the *S. intermedia* EO. It is used as a flavoring agent in cosmetic products, creams, soaps and perfumes, as well as in food items such as ice creams and fruit juices [47]. Limonene possesses bacteriostatic [48], antifungal [49] and bactericidal activities [50]. Oyedemi *et al.* [43] demonstrated the protein leakage of three typical EO components, eugenol, γ-terpinene and α-terpineol, in both Gram-positive and Gram-negative bacteria, due to cell membrane disruption.

Therefore, although the mechanism of antimicrobial activity of terpenes is not entirely known, it seems that these lipophilic compounds may alter the structural and functional integrity of the cell membrane in Gram-negative and Gram-positive bacteria as well as in fungi. Finally, additive and/or synergistic effects of the components of an EO may maximize its biological activities.

## 3. Experimental Section

### 3.1. Plant Material and Essential Oil Extraction

The aerial parts of *Satureja intermedia* C.A.Mey (*Lamiaceae*) were collected, in July 2014, at full flowering stage, from wild plants in the mountains of Sepidan (coordinates: 30°10′N–52°00′E), Sepidan County, Fars Province, Iran. The plant was taxonomically recognized by a botanist at the Herbarium of Pharmacognosy, Department of the Faculty of Pharmacy affiliated to Shahid Beheshti University of Medical Sciences of Iran. The aerial parts of the plants were dried in the shade for 72 h. For *S. intermedia* essential oil (EO) preparation, the dried aerial parts (leaves, stems and flowers) (100 g) were hydrodistilled for 4 h utilizing an all-glass Clevenger-type apparatus in accordance with the method outlined by the British Pharmacopeia [51]. The *S. intermedia* EO obtained was dried over anhydrous sodium sulphate (Sigma-Aldrich, St. Louis, MO, USA) and stored at 4 °C for gas chromatography-mass spectrometry (GC–MS) analysis and biological assays.

### 3.2. Gas Chromatography and Gas Chromatography Coupled to Mass Spectrometry Analyses

Gas chromatography (GC) analysis was carried out using a Shimadzu GC-9A gas chromatograph (Kyoto, Japan), equipped with a DB-5 fused silica column (30 m × 0.25 mm i.d., film thickness 0.25 µm). Oven temperature was performed as follows: 50 °C for 5 min; 250 °C at a rate of 3 °C/min, injector temperature and detector (FID) temperature 290 °C; helium was used as carrier gas with a linear velocity of 32 cm/s.

The gas chromatography coupled to mass spectrometry (GC–MS) analysis was carried out using Varian 3400 GC–MS system equipped with a DB-5 fused silica column (30 m × 0.25 mm i.d.). Oven temperature was 40–240 °C at a rate of 4 °C/min; transfer line temperature 260 °C; carrier gas helium with a linear velocity of 31.5 cm/s; split ratio 1/60; ionization energy 70 eV; scan time 1 s and mass range of 40–300 amu. Retention indices (RI) were determined using retention times of *n*-alkanes that were injected after the essential oil under the same chromatographic conditions. Compounds were identified by comparison of mass spectral fragmentation patterns and retention indices (HP-5) with Wiley 7n.L Mass Spectral Library (Wiley, New York, NY, USA), Adams Library and Mass Finder 2.1 [52,53,54]. The relative percentages of the components of the EO were obtained according to the peak area in the chromatogram [55].

### 3.3. Human Cancer Cell Lines

The Hep-G2 (human hepatocellular carcinoma) cell lines (ATCC^®^ HB8065™) and MCF-7 (human breast adenocarcinoma) cells (ATCC^®^ HTB22™) were purchased from the American Type Culture Collection (ATCC, Rockville, MD, USA) and used for cytotoxicity assay. The cells were cultivated in Dulbecco’s modified Eagle’s Medium supplemented with 10% heat-inactivated fetal bovine serum, 1% l-glutamine, HEPES (*N*-2-hydroxyethylpiperazine-*N*-2-ethane sulfonic acid) buffer and 50 μg/mL gentamicin (Sigma-Aldrich, St. Louis, MO, USA). The cells were stored at 37 °C in a humidified atmosphere with 5% CO_2_ and were sub-cultured three times a week.

### 3.4. Cytotoxicity Test

Cytotoxicity of *S. intermedia* EO on cancer cells was evaluated by the crystal violet staining method described by Saotome *et al.* [56] with slight modifications. In brief, the cells were incubated in 96-well tissue culture microplates (1 × 10^4^ cells per well in 100 μL of growth medium). Fresh medium containing different concentrations of the *S. intermedia* EO was added after 24 h of seeding at 37 °C. Serial two-fold dilutions of the *S. intermedia* EO were added to confluent cell monolayers into 96-well microtiter plates by a multichannel pipette. The microplates were incubated at 37 °C in a humidified incubator with 5% CO_2_ for 48 h. The viable cells were determined using a colorimetric method. Briefly, medium was aspirated and a 1% (*v*/*v*) crystal violet solution in methanol was added to each well. After 45 min, the stain was removed and the plates were carefully rinsed with distilled water. Then, 0.2 mL of glacial acetic acid-ethanol mixture (1.0 mL glacial acetic acid in 100 mL 50% ethanol) to all wells and mixed thoroughly. The absorbance was determined at 595 nm by an automatic microplate reader. The concentration at which the growth of cells was inhibited to 50% of the control (IC_50_) was calculated by using the formula previously reported by Sharifi-Rad *et al.* [8]. The control cells were incubated without test sample and with or without dimethylsulfoxide (DMSO). In this study, vinblastine sulfate was used as standard anticancer drug.

### 3.5. Antimicrobial Activities

#### 3.5.1. Microorganisms

Antimicrobial activity of the *S. intermedia* EO was assayed on oral pathogens including: *Streptococcus mutants* (ATCC 25175, ATCC 31383 and ATCC 35668), *Streptococcus salivarius* (ATCC 13419 and ATCC 9222), *Enterococcus faecalis* (ATCC 29212 and ATCC 11700), *Staphylococcus aureus* (ATCC 25923 and ATCC 700698), *Candida albicans* (ATCC 13803 and ATCC 10261) and *C. glabrata* (ATCC 2001 and ATCC 90030) were determined.

#### 3.5.2. Determination of Minimum Inhibitory Concentration, Minimum Bactericidal Concentration and Minimum Fungicidal Concentration

Minimum inhibitory concentration (MIC), minimum bactericidal concentration (MBC) and minimum fungicidal concentration (MFC) were determined using microdilution test according to Clinical and Laboratory Standards Institute with some modifications [57]. Briefly, to determine antifungal activity, serial dilutions of the *S. intermedia* EO (80–0.625 mg/mL) were prepared in 96-well microtiter plates with RPMI-1640 media (Sigma, St. Louis, MO, USA) buffered with MOPS (3-morpholinopropane-1-sulfonic acid) (Sigma, St. Louis, MO, USA). For the determination of the antibacterial activity, serial dilutions of the *S. intermedia* EO (80–0.625 mg/mL) in Muller–Hinton Broth media (Merck KGaA, Darmstadt, Germany) were prepared. Bacterial or fungal strains were suspended in the media and the cell densities were adjusted to 0.5 McFarland standards at 530 nm (this yields stock suspension of (1–1.5) × 10^8^ cells/mL for bacteria and (1–5) × 10^6^ cells/mL for fungi). Then, 100 μL of the inoculums were added to the microtiter plates which were incubated for 24 h in a humid atmosphere at 37 °C for bacteria, and for 24–48 h at 30 °C for fungi.

As sterility control, 200 μL of not inoculated medium were included. Growth controls were media with inoculums but without *S. intermedia* EO. The growth in each well was compared with that of the growth in the control well. The MICs were visually detected in comparison with the growth in the control wells and defined as the lowest concentrations of the *S. intermedia* EO producing no visible growth. Finally, media from wells with bacteria and fungi showing no visible growth were further cultured on Sabouraud Dextrose agar (Merck) to determine the MBC and MFC. These are defined as the lowest concentrations of the *S. intermedia* EO, which correspond to 99.9% mortality of the microorganisms in the initial inoculums. The number of surviving microbial cells was determined by viability counts. Vancomycin and Ketoconazole were the positive controls for bacteria and fungi, respectively.

### 3.6. Statistical Analysis

The *S. intermedia* EO were extracted in triplicate for chemical composition, antimicrobial tests and cytotoxicity screening. All the results were subjected to analysis of variance (ANOVA), following a completely random design to determine the least significant difference (LSD) at *p* < 0.05 using SPSS v. 11.5 (IBM SPSS, New York, NY, USA).

## 4. Conclusions

Even if local people still exploit native plants or plant products, based on their traditional knowledge, to treat a number of diseases, the information on the action mechanisms of these ethnomedicines and their bioactive components is still scant. Anyway, these remedies have recently received the interest of scientists and pharmaceutical industries, as a source of new, alternative, promising and, possibly, low-cost active ingredients to be developed. In these terms, the essential oil of *S. intermedia* has the potential of fulfilling these expectations, by virtue of its anticancer activity, though further preclinical studies are needed in order to assess its efficacy and safety.
